# In-Depth Investigation into the Transient Humidity Response at the Body-Seat Interface on Initial Contact Using a Dual Temperature and Humidity Sensor

**DOI:** 10.3390/s19061471

**Published:** 2019-03-26

**Authors:** Zhuofu Liu, Jianwei Li, Meimei Liu, Vincenzo Cascioli, Peter W McCarthy

**Affiliations:** 1 The higher educational key laboratory for Measuring & Control Technology and Instrumentations of Heilongjiang Province, Harbin University of Science and Technology, Harbin 150080, Heilongjiang, China; 13904502205@163.com; 2 Department of Obstetrics and Gynecology, the Second Affiliated Hospital of Harbin Medical University, Harbin 150081, Heilongjiang, China; mm7723@163.com; 3 Murdoch University Chiropractic Clinic, Murdoch University, Murdoch 6150, Australia; v.cascioli@murdoch.edu.au; 4 Faculty of Life Science and Education, University of South Wales, Treforest, Pontypridd CF37 1DL, UK; peter.mccarthy@southwales.ac.uk

**Keywords:** humidity, transient response, body-seat interface, thermal impact, sitting rate, dual temperature-humidity sensor

## Abstract

Relative humidity (RH) at the body-seat interface is considered an important factor in both sitting comfort and generation of health concerns such as skin lesions. Technical difficulties appear to have limited research aimed at the detailed and simultaneous exploration of RH and temperature changes at the body-seat interface; using RH sensors without the capability to record temperature where RH is recorded. To explore the causes of a spike in RH consistently produced on first contact between body and seat surface, we report data from the first use of dual temperature and RH (HTU21D) sensors in this interface. Following evaluation of sensor performance, the effect of local thermal changes on RH was investigated. The expected strong negative correlation between temperature and RH (R^2^ = −0.94) supported the importance of considering both parameters when studying impact of sitting on skin health. The influence of sensor movement speed (higher velocity approach: 0.32 cm/s ± 0.01 cm/s; lower velocity approach: 0.17 cm/s ± 0.01 cm/s) into a static RH region associated with a higher local temperature were compared with data gathered by altering the rate of a person sitting. In all cases, the faster sitting down (or equivalent) generated larger RH outcomes: e.g., in human sitting 53.7% ± 3.3% RH (left mid-thigh), 56.4% ± 5.1% RH (right mid-thigh) and 53.2% ± 2.7% RH (Coccyx). Differences in size of RH change were seen across the measurement locations used to study the body-seat interface. The initial sitting contact induces a transient RH response (duration ≤ 40 s) that does not accurately reflect the microenvironment at the body-seat interface. It is likely that any movement during sitting would result in similar artefact formation. As a result, caution should be taken when investigating RH performance at any enclosed interface when the surfaces may have different temperatures and movement may occur.

## 1. Introduction

Sedentary behaviour has increasingly become the norm in many societies due to the increasing reliance on technology [1]. According to previous reports [2,3,4], it has been estimated that the average adult sits for up to two thirds of their time awake. However, extensive sitting postures along with low energy expenditure (physiological inactivity) have been strongly associated with many health problems such as obesity, type 2 diabetes, musculoskeletal symptoms and cardiovascular disease [5].

Another group that can be affected by too high humidity at the seat surface-skin interface is wheelchair users. This group often suffers from various skin diseases due to prolonged mechanical loading and in some cases, their inability to regulate air circulation in order to maintain their skin’s viability. A typical and severe problem is the formation of pressure ulcers (PUs) when the skin is subjected to a mixture of persistent pressure, high humidity, raised metabolism and reduced blood flow. The yearly expenditure on PU diagnosis and treatment imposes heavy burdens on the health care sector. Between 1990 and 2001, it was reported that 114,380 people died from PU-related illnesses in the United States [6,7] alone. Although the cause of PUs is multifactorial, prolonged moisture and mechanical loading have been considered the main factors underpinning their formation and development [8].

Regarding the relationship between PUs and moisture build-up at the skin-seat/skin-mattress contact surface, a mathematical model was established to optimise the microclimate factors including relative humidity (RH), temperature (T) and pressure (P) [9]. From the perspective of aetiology, the causation of PUs in chronically immobilised patients was investigated and the results indicated that ensuring the flow of water vapour from the patient’s body to the outside was an essential component in the prevention of skin lesions [10,11]. RH has been identified as a primary cause of PU formation together with lack of activity, friction and shear [12]. As there are no obvious symptoms prior to the occurrence of PUs, monitoring the microclimate characteristics at body-seat interface may be an effective tool to help reduce its prevalence.

A number of developments have been reported in order to allow the testing of designs, including a sitting simulator to evaluate the microenvironment properties of wheelchair cushions [13] and a personalised seat ventilation system capable of compensating for the RH deficit in a relatively high-density space (aircraft cabins [14] or classrooms [15]). To evaluate the performance of moisture dissipation, the TRCLI (thermodynamic rigid cushion loading indenter) was developed and used to compare the capabilities of different wheelchair cushions in terms of dissipating water vapour [16]. Furthermore, wheelchair cushion selection criteria have been proposed taking the transfer of water vapour into consideration [17].

Although RH has been recognised as playing an important role in the formation and development of PUs, it has not proved easy to measure reliably without the researcher having to directly influence the environment and can be prone to be influenced by the adjacent environment. We have performed sitting experiments in a wide range of ambient conditions (damp summer days in the UK and very dry cold winter days in China) [18,19]. Although experimental room conditions were similar in terms of temperature, obvious differences existed in ambient moisture and, as a result, background RH. Regardless of these differences, the profile of RH change on sitting and remaining seated generally appeared very similar. Although much of the experiment produced expected results, one segment of the RH sensor output regularly caused us to pause for thought, namely the initial recording period. This period focuses on the duration directly related to sitting down and was associated with an apparent spike in RH [18]. In previous recordings, we were using humidity sensors without any direct temperature assessment. Although we had recognised the spike to most likely be an artefact related to our methodology, another researcher in the field (Siyu Lin) reported seeing a similar artefact. On raising the question of cause, we decided to explore the transient response of RH characteristics at the contact surface. The rationale for this was two-fold. Firstly, to understand the changes occurring at this point and attempt to describe any artefact components as they may mask other potentially important data; secondly, to ensure an understanding of why this occurred as it might also be capable of affecting recordings made later in any sitting session. Fortunately, the timing of this research allowed us to be first to report the use at the skin-seat interface of a sensor chip, which had both RH and temperature measurement capability. This chip gave us, for the first time, the opportunity to accurately co-locate these two measurements and, therefore, look to the degree of relationship between them. Therefore, the purpose of this study was to offer comprehensive insight into the RH varying patterns over time following the user’s initial contact with the seat.

## 2. Materials and Methods

### 2.1. Hardware System Description

The main component of the data acquisition unit is an ATmega328 processor (Microchip Technology, Chandler, AR, USA) which is connected to the HTU21D sensors (capable of simultaneously acquiring RH and temperature information: TE Connectivity Ltd., Rheinstrasse, Schaffhausen, Switzerland) through an IIC (Inter-Integrated Circuit) interface. Collected data are transferred from the processor to the computer through a USB cable and stored in the computer’s hard drive disk for further off-line analysis. The sampling interval chosen is 10 Hz as the rate of temperature and RH change at the body-seat interface is much lower than this frequency [18,19].

### 2.2. Sensor Evaluation

Prior to conducting any experiment, a freshly acquired, commercially available HTU21D sensor randomly selected from a recently purchased package was assessed to determine its performance, particularly accuracy and linearity along with repeatability and hysteresis. A standardised environmental chamber (PVS-3KP, ESPEC Environment Equipment Co. Ltd., Hudsonville, MI, USA) was used for this purpose, having the capability of providing reliable RH variations from 10% RH to 95% RH (Certificate No: ISO 04308Q11746R0 M and EN AC/0708030).

The RH range of the chamber was manually changed to 13–93% RH, with increment/decrement steps of 4% RH. The chamber temperature was set to 25 °C ± 0.1 °C, which is the recommended testing temperature for the sensor in accordance with the manufacturer’s data sheet. At each testing point, the stabilised sensor’s output was recorded and comparative tests were carried out using the averaged value of five outputs (at each stabilised testing point) from the sensor. In addition, RH increment/decrement tests were performed five times in order to examine the repeatability.

### 2.3. Consistency Test

Three HTU21D sensors were attached to the contact surface of the foam cushion in the approximate locations suggested by previous research [18,19]. Briefly, these are: one on each side of the cushion symmetric to the central line (approximating the middle of each thigh) and a further sensor at the rear (at a point approximating the location of the coccyx region). To replicate the influence of pressure imposed on sensors due to human sitting, dummy buttocks were created. They were made by inserting sand bags into a pair of jeans with the lower legs sewn shut. The total mass of the calibration dummy buttocks was 50 kg in order to mimic the human loading introduced to cushions used in previous studies [19,20,21].

Attention was paid to ensure that all the sensors would be fully covered by the dummy buttocks in the loading trial. A 1-h duration trial was conducted in a vacant research room with environmental conditions: 27.8 °C ± 0.1 °C and 39.9% ± 0.2% RH. The door of the research room was closed during the entire experiment with no researchers remaining in the room during this time. This precaution was to limit the likelihood of any possible disturbance to the room temperature and RH levels.

### 2.4. Heating Trial

To examine the influence of thermal variations on the sensors RH output, an experiment was carried out to imitate body-seat interface RH changes at the initial point of sitting down under the environmental condition: 43.0% ± 1.4% RH and 22.5 °C ± 0.2 °C. An HTU21D sensor was placed in a cavity (63.3 × 40.4 × 18.7 mm: Length, Width and Depth, respectively) cut into the surface of a foam seat pad (cushion). An aluminium water tank (223 × 132 × 66 mm: Length, Width and Depth, respectively) containing half its total volume of tap water was then positioned on the top of this slot. Water temperature was monitored by reading from an Hg thermometer in the water tank. Boiled water was gradually added to the water already in the tank until the water temperature in the tank reached 40 °C. The water was then allowed to cool until the temperature in the water tank dropped to around 20 °C. During the whole process, both temperature and RH values inside the slot in the foam cushion were recorded by the HTU21D sensor and transmitted from the digital communication interface of the microprocessor to the computer through a USB cable.

### 2.5. Human Trial

A healthy university student (174 cm and 58 kg) voluntarily participated in all tests, which had been approved by the ethics committee affiliated to Harbin University of Science and Technology. The participant gave written informed consent prior to volunteering. To prevent any effect caused by the material of any garment worn, the participant was asked to wear jeans while taking part in all trials. Measurements were made in the same laboratory as the other tests with both ambient temperature and RH being continuously monitored. The laboratory door was closed during the testing period to avoid any disturbance to the environmental conditions.

#### 2.5.1. Sensor Movement

**Figure 1:**
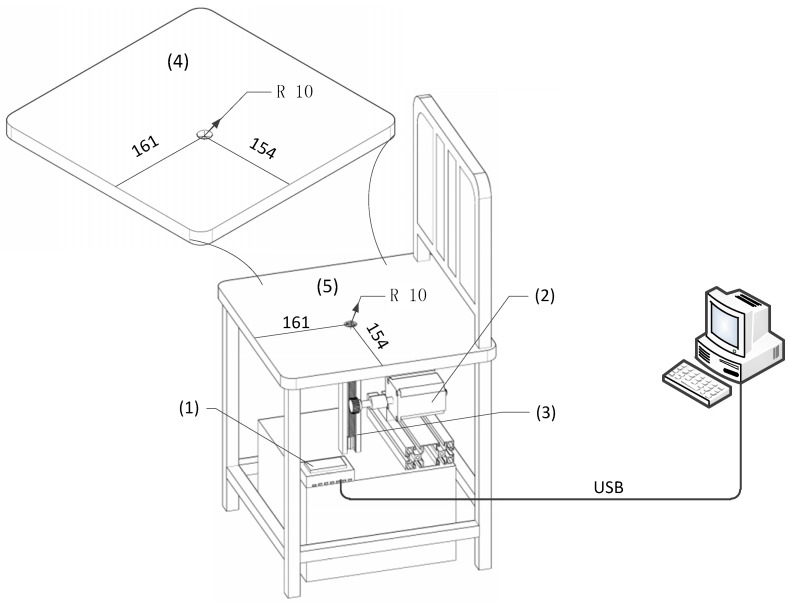
Configuration of the sensor movement system. Major components included: (1) data acquisition unit, (2) stepping motor, (3) transportation mechanism, (4) custom-made foam cushion with a drilled hole and (5) the wooden chair surface configured with the same hole. Both the customised cushion and the chair had equal dimensions (399 × 378 mm, length and width, respectively).

Presuming that differences in approach speeds between body and seat interface could have an impact on the transient RH variance, an adjustable speed system (Figure 1) was developed on which the sensor was mounted. During the trials, the body was kept in contact with the seat surface (relative static) while the sensor approached the contact surface at different speeds. The sensor was not fixed in the hole for the following reasons: (1) approach velocities between body and sensor would not be consistent between each trial; (2) the sensor’s movement was considered equivalent to the movement of the participant according to relative movement theory [22]. Additionally, it was possible to adjust the moving speeds precisely by programming the microprocessor. 

Based on the above design considerations, the adjustable speed system consists of a stepping motor, a rack and pinion, a control unit based on ATmega328 processor (Microchip Technology, Chandler, AR, USA) and some peripheral circuitry. The HTU21D sensor was fastened to a custom-made rack and pinion platform using hot melt adhesive (Delixi Electric Ltd., Zhejiang, China). A hole (20 mm in diameter) was created in a foam cushion fixed on a commercially available wooden chair. The hole was made at approximately the position that the middle part of the left thigh would be expected to occupy in a seated person. The hole pierced the whole of the foam and seat below. The sensor was driven upwards through the hole from below the chair towards the uppermost surface of the foam.

Testing was divided into two trials according to the sensor’s movement speed (0.32 cm/s ± 0.01 cm/s and 0.17 cm/s ± 0.01 cm/s). Each trial was repeated 10 times, with a 20-min recovery interval being provided between each trial in order to ensure the sensor had similar starting conditions. As previously, in order to reduce the impact of environmental changes, the whole test was completed in the same laboratory, with the door closed throughout the recording period. The ambient temperature and RH of the laboratory during these trials were 28.5 °C ± 0.2 °C and 57.9% ± 0.9% RH, respectively.

After initialising the system, the participant was asked to take a seat. The sensor system was then programmed to move up through the hole at the preset speed. When the sensor came into contact with the left mid-thigh (measurement position), the stepping motor stopped. During the whole process, RH values were recorded and the last five recordings were averaged to represent the immediate contact RH value.

#### 2.5.2. Participant Movement

In this part of the trial, three HTU21D sensors were fixed to the foam cushion at three sensitive locations described above: left mid-thigh, right mid-thigh and coccyx relative to the body structure of the participant [18]. To evaluate the effect of sitting speed on the interface RH, the same participant sat down at two different speeds (slowly or rapidly). Firstly, the participant stood in front of the test chair and waited for the order to sit down. The data acquisition system was activated to record the initial RH values, which were used as reference baselines. At this point, the participant was asked to “take a seat” (either slowly or quickly). After the participant sat properly (the buttocks were fully in contact with the foam cushion), the data acquisition process was manually terminated. The sitting speed trials were repeated ten times with a 20-min time gap between trials to allow sensors to recover to a condition similar to that at the start. The ambient temperature and RH parameters during these experiments were 24.7 °C ± 0.2 °C and 38.6% ± 0.4% RH, respectively.

### 2.6. Statistical Analysis

Normality of the data was assessed using Kolmogorov-Smirnov tests followed by appropriate paired test (t-test) to investigate the influence of sitting speed on RH at the sensor site. One-way analysis of variance (ANOVA) was used to examine RH characteristics among different measurement locations (participant movement trials), followed by a post-hoc Tukey-Kramer test [23] where appropriate. Along with the ANOVA, a Tukey-Kramer test is effective at determining the difference among each series of reading by comparing all possible pairs of means. The significance level was set as p < 0.05 for all statistical analyses. Finally, correlation analyses were performed to determine potential relationships between obtained measures.

## 3. Results

### 3.1. Sensor Performance

**Figure 2:**
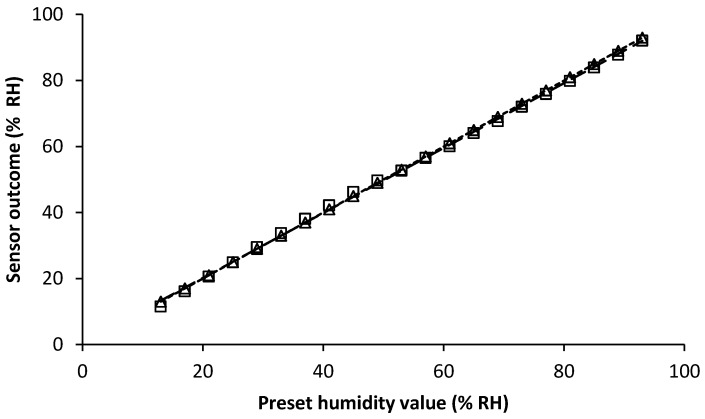
Performance of the humidity component (RH) of the HTU21D sensor. The square shape (□) indicates the measurements associated with increments in RH while the triangle shape (△) indicates measurements associated with decrement changes. The dashed line is the fitting curve for the increment data and the dotted line is the fitting curve based on the decrement data. The fitting equation for the increment test is y = 0.984x + 0.5124 while for the decrement test it is y = 1.0111x + 0.4142.

Figure 2 illustrates the sensor’s performance in terms of linearity, repeatability and accuracy using data from the environmental chamber trials where RH was increased and then decreased, at the same temperature. Regarding accuracy, the absolute maximum difference between average (n = 5) reported value and the standardised RH values (environmental chamber output) was 1.8% RH over the full testing range. Regarding repeatability, the absolute maximum deviation among the five tests was 0.5% RH.

Output from the sensors also exhibited an approximately linear relationship in relation to that from the standardised environmental chamber (R^2^ = 0.9989 and 0.9987 for increasing and decreasing RH trials, respectively). In terms of hysteresis resulting from increasing and decreasing RH, the correlation between RH outputs from the sensors was R^2^ = 0.9997.

**Figure 3:**
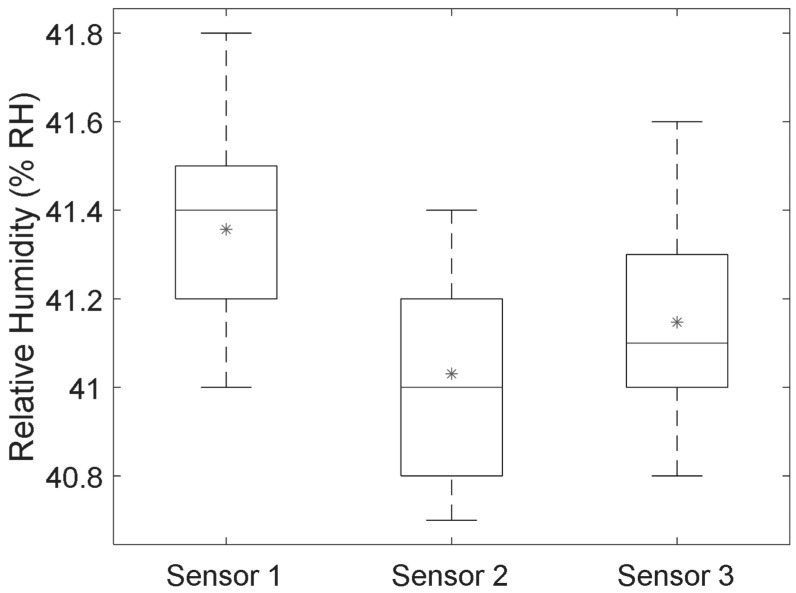
Box and Whisker plot of the data from the consistency test for the three humidity sensors using dummy buttocks to imitate the body pressure on the cushion surface. The experiment was performed in a vacant laboratory over a period of one hour (environmental temperature and RH: 27.8 °C ± 0.1 °C and 39.9% ± 0.2% RH). Top and bottom whiskers on the figure represent the maximum and minimum values for the corresponding humidity sensors, while the line inside each box represents the median value. The upper and lower borders of the boxes represent the 75th and 25th percentile values, respectively. In addition, the average values have been indicated by ‘*’ in the boxplot.

As three sensors were to be used in trials, their outputs were compared (Figure 3) under a sitting simulator (to simulate static loading pressure of sitting) and in a constant humidity/temperature environment for 1 hour. Outputs from the three sensors (Mean ± 1SD) were 41.4% ± 0.2% RH, 41.0% ± 0.2% RH and 41.1% ± 0.2% RH, respectively. A one-way ANOVA revealed no significant difference between the three sensors (p > 0.1).

### 3.2. Relationship between Temperature and RH

**Figure 4:**
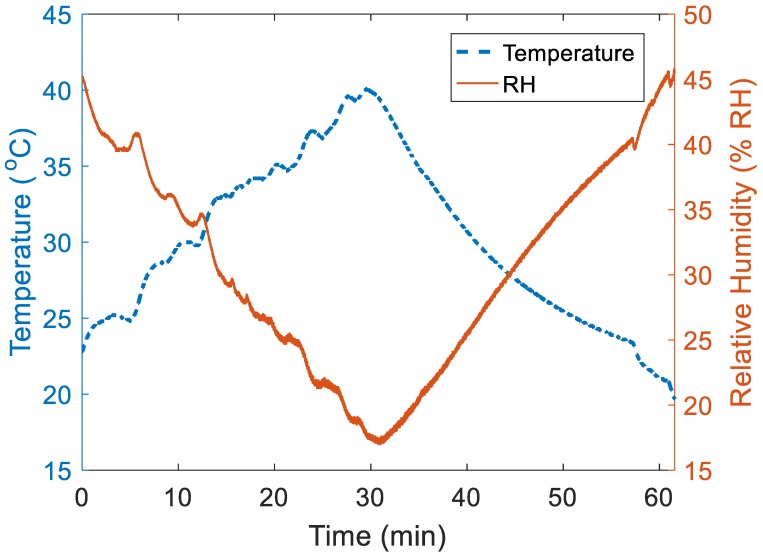
Graphical representation of the one-hour heating trial to illustrate the relationship between temperature change and relative humidity (RH) inside the relatively sealed environment created within the slot in the foam cushion. The dashed line represents the variation in temperature values (created by changing water temperature inside a metal container, which was monitored by reading from an Hg thermometer) and the solid line is the response of RH within the enclosed space in the foam cushion slot (environmental condition: 43.0% ± 1.4% RH and 22.5 °C ± 0.2 °C).

The influence of temperature changes on RH within a small region was assessed under the following environmental conditions: 43.0% ± 1.4% RH and 22.5 °C ± 0.2 °C (Figure 4). During the heating stage (hot water filled into the tank causing the air temperature inside the slot of the foam cushion to steadily rise due to thermal exchange), RH values within the region dropped to the lowest points (17.2% RH). In the natural cooling stage (the water was left to cool down without any interference), RH values gradually increased and stabilised at 45.2% RH. The correlation coefficient between temperature and RH value was −0.94.

**Figure 5:**
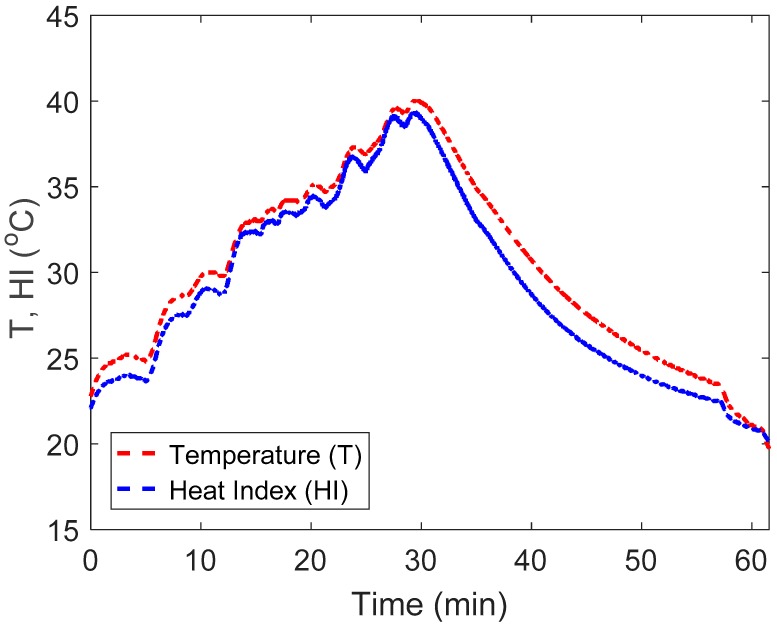
Comparison of heat index (HI) and measured temperature (T), both reported as °C, where the red line represents the measured temperature values and the blue line is HI calculated using relative humidity (RH) and the temperature from within the slot in the foam cushion.

To further analyse the relationship between the temperature and RH (Figure 5), Heat Index (HI) was calculated using the following equation [24]:

(1) $$HI=c_{1}+c_{2}T+c_{3}R+c_{4}TR+c_{5}T^{2}+c_{6}R^{2}+c_{7}T^{2}R+c_{8}TR^{2}+c_{9}T^{2}R^{2}$$

where:c_1_ = 0.363445176,c_2_ = 0.988622465,c_3_ = 4.777144035c_4_ = −0.114037667,c_5_ = −8.50208 × 10^−4^,c_6_ = −2.0716198 × 10^−2^c_7_ = 6.87678 × 10^−4^,c_8_ = 2.74954 × 10^−4^,c_9_ = 0

To examine how RH varies according to the changing temperature, Dew Point (DP) values were also calculated [25]:

(2) $$\mathrm{DP}=\frac{243.04\times \left(\ln\left(\frac{\mathrm{RH}}{100}\right)+\frac{17.625T}{243.04+T}\right)}{17.625-\ln\left(\frac{\mathrm{RH}}{100}\right)-\frac{17.625T}{243.04+T}}$$

The calculated DP values were compared to a DP table (https://www.lamtec.com/technical-bulletins/dew-point-table/) utilising three testing points (starting point, near peak and end point) to examine RH variation in relation to changes in temperature.

### 3.3. Measurement of the Body-Seat Interface RH

#### 3.3.1. Sensor Movement

**Figure 6:**
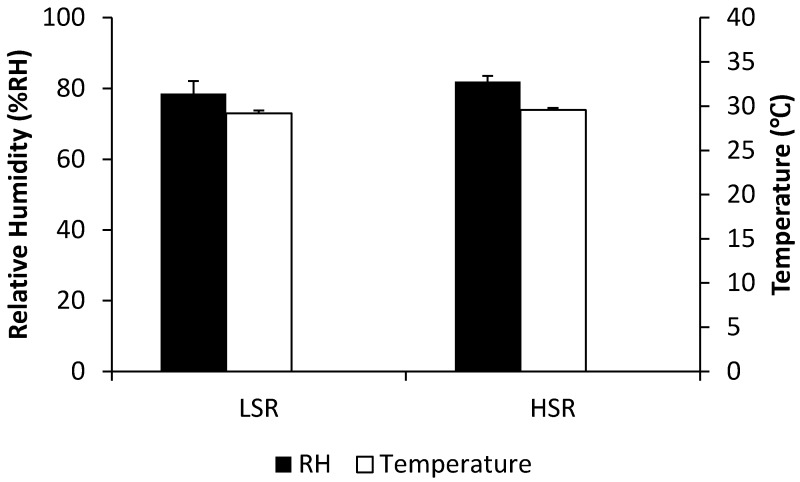
Comparison of body-seat interface relative humidity (RH) when the sensor moves towards the left mid-thigh of the participant at two different velocities: lower speed rate (LSR; 0.17 cm/s ± 0.01 cm/s) and higher speed rate (HSR; 0.32 cm/s ± 0.01 cm/s). In addition, the temperature values at the contact surface for different speeds of sensor movement are illustrated along with RH. Error bars denote ±1 standard deviation.

In the sensor movement tests (n = 10), significantly different (p < 0.05) RH values at the body-seat interface were found between the fast (85.0% ± 1.6% RH) and slow (78.5% ± 3.5% RH) velocity of the stepping-motor-driven sensor system (Figure 6). 

**Figure 7:**
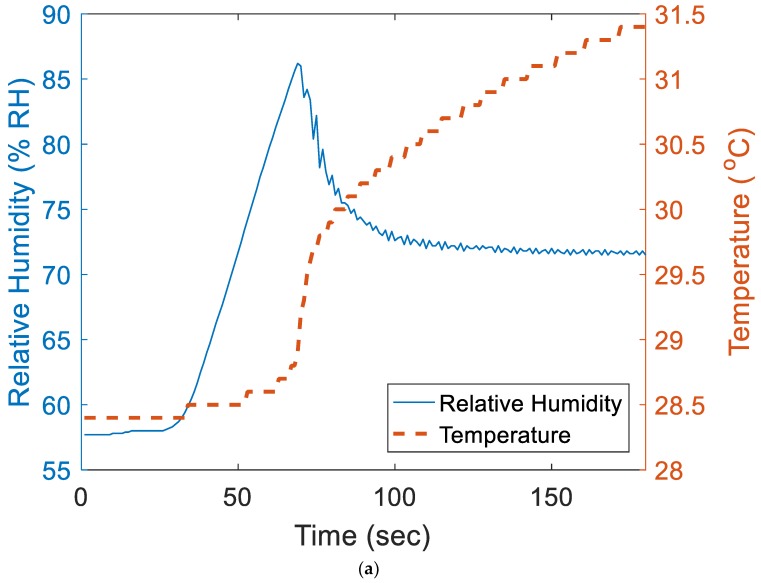

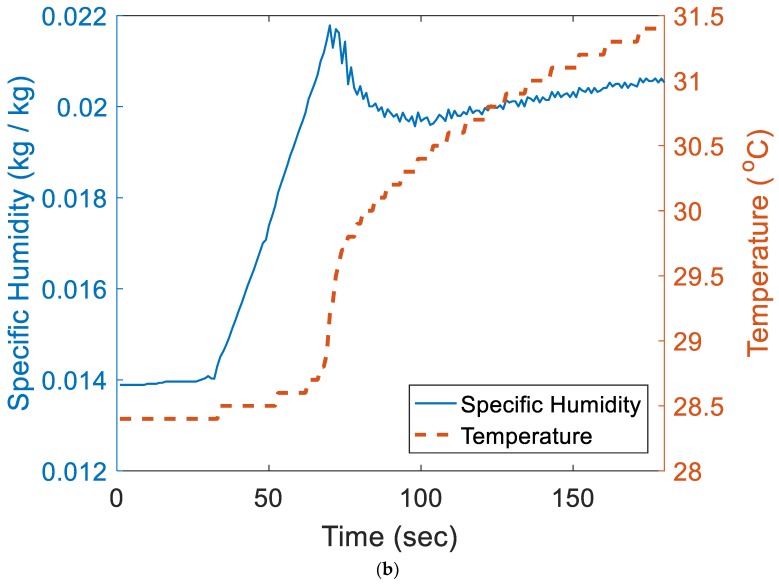
Varying patterns of temperature and moisture based on a data set from one of the 10 repeat trials. After the sensor was fully in contact with the mid left-thigh of the participant, the system remained in that position to record information for approximately 100 seconds: (**a**) relative humidity (RH) and temperature (T); (**b**) specific humidity (SH) and temperature where SH data were estimated using a humidity converter from the following website (http://www.humcal.com/index.php).

To analyse the relationship between the sensor approach speeds and RH, the average values of each trial for sensor speed and its relative RH were assessed for the presence of a correlation. However, no correlation was observed between high speed and RH, whereas at best a weak correlation might exist with slow speed (correlation coefficients 0.02 and 0.64, respectively). In addition, the trend curve for both moisture and temperature showed an apparent “spike” after the sensor achieved full contact with the left mid-thigh of the participant (Figure 7).

#### 3.3.2. Participant Movement

**Figure 8:**
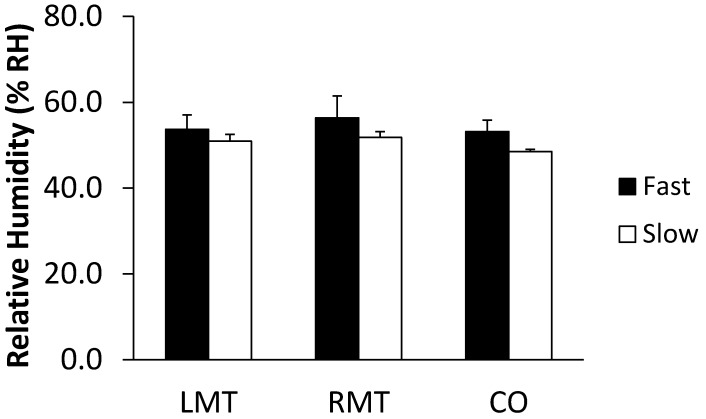
Averaged relative humidity (RH) values based on 10 sets of measurements including the last five measuring points from each of the three sensitive locations: left mid-thigh (LMT), right mid-thigh (RMT) and coccyx (CO), when the participant sat down at different speeds (fast: 10.29 ± 0.90 cm/s; slow: 6.78 ± 0.43 cm/s). Error bars denote the first standard deviation. The ambient temperature and RH parameters were 24.7 °C ± 0.2 °C and 38.6% ± 0.4% RH, respectively.

When the participant sat down either slowly or rapidly, the body-seat interface RH responded differently at the measurement locations (Figure 8). Based on the 10 repeat trials, the statistical values (Mean ± 1SD) were 51.0% ± 1.6% RH (left mid-thigh), 51.8% ± 1.3% RH (right mid-thigh) and 48.5% ± 0.5% RH (Coccyx) for sitting slowly. In comparison, the fast sitting down generated larger RH outcomes: 53.7% ± 3.3% RH (left mid-thigh), 56.4% ± 5.1% RH (right mid-thigh) and 53.2% ± 2.7% RH (Coccyx).

Significant differences were found among different sitting speed for each measurement location (p < 0.05: paired t-tests). The sitting speed in the sagittal plane was estimated by measuring the vertical and horizontal distances between the bottom (initial contact point) just before sitting and the contact point on the seat cushion. The trajectory between these two points was estimated by assuming a right angled triangle and calculating the hypotenuse in order to approximate the moving path during sitting down process (linearity was assumed for ease of calculation, although it is acknowledged that the path was likely to be slightly curvilinear). The time taken to sit for each participant movement trial was recorded and divided by the estimated distance travelled. 

Another interesting discovery was that significant differences also existed among the different measurement locations at the lower sitting speed (p < 0.05), while the difference was not significant for the faster sitting speed (p = 0.13). To further explore this apparent difference, a Tukey-Kramer test was applied to analyse the RH data from the slow sitting down experiments. Results indicated that the coccyx region sensor showed a significant difference from both mid-thigh measurements (the difference between left mid-thigh and coccyx was 2.4% ± 1.5% RH and it was 3.3% ± 1.5% RH between right mid-thigh and coccyx), while there was no significant difference between left and right mid-thighs.

## 4. Discussion

### 4.1. Sensor Evaluation

Based on the sensor performance tests, it is conceivable that HTU21D is more suitable for detecting body-seat interface microenvironment parameters than traditionally used single modality sensors [18,19,20]. Firstly, the accuracy is ± 2% RH along with lower hysteresis (±1% RH) according to the sensor’s datasheet, confirmed in part by the findings presented here. Secondly, it integrates both temperature and RH detectors in a single tiny microelectronic chip (3 × 3 mm). As a result, it is possible to measure both thermal and humidity information at the same contact area without the need of deploying two solo-functioning (temperature/humidity) transducers. The thermal detection range is from −40 °C to 100 °C with the capability for full range RH measurement (0% RH to 100% RH). Lastly, but not least, the price for a breakout board of HTU21D is minimal, making it financially and practically possible to construct a sensing array for use at the contact surface similarly to our previously published research [20].

### 4.2. Transient Characteristics of RH at the Contact Surface

Based on the experimental results presented here, we conclude that temperature variance can and does influence the recorded RH during the initial period when the surfaces move into contact A further factor appears to be the speed of the sensor approaching the buttocks and vice versa (i.e., speed of sitting down). RH is directly related to the temperature of the sensor when it makes the reading; therefore, knowledge of the temperature would be considered important. When two surfaces at the same RH but different temperatures come into contact, the sensors may misreport the water vapour before the temperatures of the surfaces become equilibrated. Hence, research would require an estimation of the period of time, which would guarantee equilibration with the environmental temperature. The application of both temperature and RH sensors within such a small space makes it possible to recognise the difference in equilibration and therefore see the RH change ahead of the temperature change. Lower sensor temperatures will result in the same proportion of water vapour in the air being interpreted as a higher RH, which was clearly shown by the heating trials (Figure 4). The faster the approach speed between the sensor and temperature source, the larger RH output generated (artefact: Figure 6 and Figure 8), probably as a result of the rather instantaneous presentation of skin surface water vapour and the more delayed transmission of skin associated temperature changes (Figure 7). The human body is a complicated thermoregulatory system that keeps core body temperature at approximately 37 °C through various mechanisms, including sweat evaporation [26]. In addition, choice of clothing material can be used to either enhance or hinder both heat and water vapour transfer out from the body, and thus by extension, will affect transfer to and from any surface the skin comes into contact with [27] and should be carefully considered for any experimental assessment of this interface, along with the surface RH characteristics [13,16]. This finding highlights the importance of not only building an integrated microenvironment (both temperature and humidity generator) simulation system but considering the impact of loading rate (e.g., sitting speed) when using either a human or a dummy to investigate RH changes at the body-seat interface [13]. 

In the heating trial, the RH reached equilibrium (Figure 4) after the water temperature (19.9 °C) in the tank had nearly returned to room temperature (22.5 °C ± 0.2 °C). This phenomenon supports the delay in the interpretation of RH by the system caused by the sensor failing to equilibrate quickly enough to changes in temperature. Hence, there will always be a prolonged period to achieve an accurate RH equilibrium (i.e., reading the accurate RH at the set temperature). The rate of change was found to be 0.9% RH/min for the whole testing process (from heating to cooling). These findings indicate that thermal conditions should be taken into account when analysing the RH variations [16] as the amount of water vapour present could also be expected to affect temperature transfer rate. This aspect has been verified with the calculations of HI which reflects the combined influence of temperature and RH (Figure 5). The rising and falling trend of calculated HI and measured temperature showed a similar pattern (difference in mean ± SD: 1.1 °C ± 0.5 °C). In addition, there was a strong negative correlation between temperature and RH. It is this association between temperature and RH that may underpin the “spike” phenomenon when measuring RH at the body-seat interface during the initial period of sitting (Figure 7). The DP tests showed that there were no obvious differences in the calculated values and previously published ones (technical bulletin Number 10, Lamtec Co., Bethel, PA, USA).

The general finding of a lack of difference between left mid-thigh and right mid-thigh is consistent with our previous reports showing the RH distribution to exhibit a symmetrical pattern when a healthy participant uses a standardised sitting posture (e.g. sitting upright without fidgeting or movement) [18]. As the thighs and coccyx are significantly different, it supports the necessity of utilising multiple sensors measurement points when investigating microenvironment characteristics over the whole contact surface. The slower rate of approach between the sensor and person sitting showed differences; however, the faster speed of approach failed to show a similar pattern (no significant difference among different measurement locations). This may be due to larger magnitude of air movement resulting in some water vapour being expelled from the small region where the sensors were placed during faster sitting process. This supports the need to pre-determine the rate of sitting and movement when deciding the protocol, as rapidity of change in position can differentially affect the size of any RH change associated with the movement.

### 4.3. Limitations

Though some apparently meaningful findings have been reported here, there were several limitations to the current study. Regarding the response time of sensors, we relied on the datasheet and previously published research results [18,19] when deciding which sampling frequency to choose. Though the typical response for the HTU21D is 5 s (Max = 10 s), it would be better to directly evaluate this performance in future work, to avoid the issues identified here related to the delay in equilibration of temperature. Then, the trials of sitting speed (either sitting down or sensor movement) were conducted consecutively (i.e., 10 repetitions at the same rate of movement), based on the consideration that the reliability and consistency between each sit down phase would more likely be higher if all the repeats of a single sitting speed were performed consecutively. However, selecting the speed randomly may have been a better solution to reduce additional issues (e.g., anxiety/boredom) associated with continuously repeating the activity at the same speed. Additionally, we assumed the movement to be linear, which may have added a slight variance based on choice of sitting movement method chosen by the subject for each test. A further limitation might have been the start/stop points of the stepping motor in the current study, which was determined by estimating the running time (assuming the distance and speed were constant values). However, it is difficult to ensure every movement was a uniform rectilinear motion due to the probably presence of mechanical and electronic errors. For more accurate measurements, a proximity sensor would have been a better solution to control the stop point of the stepping motor and allow more accurate determination of the end-point distances in this enclosed space. It might also be useful to repeat the study with more participants in order to verify the universality of the findings.

Notwithstanding the aforementioned weaknesses, the strengths of this study appear obvious:

1. Evaluate the performance of the temperature-humidity-integrated sensor and determine the potential for (and confounding factors underpinning) the artefact based changes in RH. Our results suggest that the HTU21D could be considered a more ideal choice for simultaneously measuring the microenvironment (both temperature and RH) changes at the body-seat interface.
2. Demonstrate that a rapid heating or cooling could have a strong impact on reported RH values owing to the environmental changes (such as thermal exchange) within a small area. It must be remembered that the body-seat interface RH has an association with the body temperature transmitted from user to sensors. As heat conduction through air is slow, the RH estimation at skin levels will be subject to artefact enhancement until the temperature of the sensor approximates that of the skin.
3. The initial sitting contact induced RH peak could be considered an artefact resulting from the increased moisture associated with the warmer body entering the small region over a colder sensor. This finding further highlights the importance of monitoring temperature changes while investigating the RH variations at the contact surface. This monitoring is not only important for the start of sitting, but also during prolonged periods of sitting as the person starts to fidget.

## 5. Conclusions

The findings support the hypothesis that the transient increase in RH at the onset of sitting is an artefact as a result of moisture from a warmer environment interacting with a colder sensor. This spike in RH occurred during the sensor movement trials (Section 2.5.1) and attained 80.2% ± 3.2% RH with a body-seat interface temperature of 29.4 °C ± 0.3 °C. Following the initial point of contact, the RH peak declined in magnitude as the temperature of the sensor increased up to the point of thermal equilibrium in that environment. The stable outcome of RH in the sensor movement trials was 65.5% ± 3.3% RH and temperature between skin surface and seat contact surface reached 32.4 °C ± 0.4 °C. Therefore, when evaluating microenvironment variations between the body and seat interface, it appears critically important for researchers to consider adapting methodological changes to limit the impact of movements when sitting and recording RH. Although RH changes are most noticeable in the initial contact period (approximately the first 40-seconds of data), it is likely that similar effects occur as the result of movements in prolonged sitting experiments. In addition, the correlation between RH and temperature is so strong that it is necessary to monitor temperature while investigating RH changes at the contact surface.
